# Virtual Reality-Based Versus Traditional Teaching Approaches in the Oral Hygiene Education of Children with Autism Spectrum Disorder

**DOI:** 10.3390/jcm14165795

**Published:** 2025-08-15

**Authors:** Antonio Fallea, Carola Costanza, Simona L’Episcopo, Massimiliano Bartolone, Francesco Rundo, Daniela Smirni, Michele Roccella, Maurizio Elia, Raffaele Ferri, Luigi Vetri

**Affiliations:** 1 Oasi Research Institute-IRCCS, Via Conte Ruggero 73, 94018 Troina, Italy; afallea@oasi.en.it (A.F.); slepiscopo@oasi.en.it (S.L.); mbartolone@oasi.en.it (M.B.); frundo@oasi.en.it (F.R.); melia@oasi.en.it (M.E.); rferri@oasi.en.it (R.F.); 2 Department of Psychology, Educational Science and Human Movement, University of Palermo, 90128 Palermo, Italy; daniela.smirni@unipa.it (D.S.); michele.roccella@unipa.it (M.R.); 3 Department of Medicine and Surgery, Kore University of Enna, 94100 Enna, Italy; 4 UOC NPIA-ASP Enna, Child Neuropsychiatry Unit of Nicosia, 94014 Nicosia, Italy; luigi.vetri@asp.enna.it

**Keywords:** virtual reality, ASD, oral hygiene, home-based education, assistive technology

## Abstract

**Background/Objectives**: Maintaining proper oral hygiene is particularly challenging for individuals with autism spectrum disorder (ASD) due to sensory sensitivities, communication difficulties, and anxiety. Traditional oral hygiene education methods may be ineffective for this population, thereby necessitating innovative solutions. This study evaluates the effectiveness of a virtual reality (VR)-based educational program in improving home oral hygiene practices among children and adolescents with ASD. **Methods**: Sixty-four children with ASD (Level 1) were recruited and divided into two groups. Group 1 received traditional oral hygiene education, while Group 2 used a VR-based intervention featuring a virtual domestic bathroom with an avatar demonstrating proper brushing and flossing techniques. The intervention lasted eight weeks, with two one-hour sessions per week. The oral health assessment tool (OHAT) was used to evaluate oral hygiene status before and after the intervention. An unpaired t-test compared outcomes between groups. **Results**: Both groups showed improvements in oral hygiene, but the VR intervention group exhibited a significantly greater reduction in OHAT scores compared to the traditional education group (*p* < 0.001) due to a greater improvement in oral health. The VR-based approach enhanced engagement and adherence to oral hygiene routines, particularly benefiting children with ASD who struggle with conventional methods. **Conclusions**: VR-based education appears to be a promising tool for improving oral hygiene habits in children with ASD by providing an interactive and immersive learning experience. Future research should explore long-term adherence and the broader application of VR in healthcare education.

## 1. Introduction

Autism spectrum disorder (ASD) is a complex neurodevelopmental condition defined by persistent deficits in social communication and interaction, alongside restricted and repetitive behaviors, interests, or activities. Its reported prevalence has risen markedly over recent decades, currently affecting approximately 1 in 44 children worldwide [1]. Many individuals with ASD also exhibit atypical sensory processing—both hypersensitivity and hyposensitivity—to tactile, auditory, olfactory, or visual stimuli. Such sensory differences can interfere with everyday self-care routines, including tooth brushing and flossing [2].

Maintaining adequate oral hygiene is essential for overall health. Yet children and adolescents with ASD face unique obstacles in developing consistent oral care habits. Sensory aversions—to the taste or texture of toothpaste, the feel of bristles, or the sound of a toothbrush motor—often provoke distress and resistance [3]. Conversely, those with sensory hyposensitivity may not perceive discomfort or recognize the need to clean their teeth, leading them to neglect daily hygiene [4].

Beyond sensory issues, behavioral rigidity, communication challenges, and executive function deficits can further impede the establishment of effective routines. Many children with ASD require substantial caregiver support and structured instruction to acquire and maintain personal hygiene skills [5,6,7]. Moreover, selective eating and diets high in carbohydrates and sugars exacerbate their risk of dental caries and periodontal disease [8]. Consequently, studies consistently report poorer oral health outcomes in individuals with ASD—higher rates of tooth decay, gingival inflammation, and unmet dental needs—compared to their typically developing peers [1,9].

Traditional oral hygiene education strategies—verbal instructions, written leaflets, or brief clinical demonstrations—often prove insufficient for children with ASD when not tailored to their needs. Instead, they benefit most from visual supports, predictable routines, task analysis approaches, and multisensory learning tools that encourage activity repetition [10,11].

In recent years, virtual reality (VR) has emerged as a promising tool in autism research. By creating immersive, interactive environments that mimic real-world contexts under controlled conditions, VR aligns well with the visual learning strengths of many individuals with ASD [12]. VR-based interventions have successfully taught social skills, promoted emotional regulation, reduced phobias, and improved daily living abilities in this population [13,14,15].

Applied to dentistry, VR has shown encouraging results in reducing anxiety and improving cooperation during dental procedures. For example, Al Kheraif et al. (2024) reported that VR exposure before and during dental treatment significantly lowered behavioral distress and enhanced compliance among children with ASD [16]. However, the use of VR as an at-home educational tool for teaching proper brushing and flossing remains largely unexplored.

A home-based VR program could simulate a familiar bathroom setting and present an avatar demonstrating precise oral care techniques [9]. Interactive feedback, repetition, and gamified rewards may boost both skill acquisition and long-term motivation, helping bridge the gap between clinical recommendations and daily behaviors [17].

## 2. Study Objectives

This study aims to evaluate the effectiveness of a VR-based oral hygiene education program for children and adolescents with ASD in a home setting. Specifically, whether or not the regular use of a tailored VR application can increase motivation and adherence to oral hygiene routines, as reflected by improved OHAT scores over time.

By focusing on a technology-driven, home-based approach, this research seeks to provide empirical evidence for an innovative method that bridges the gap between clinical dental recommendations and daily oral care practices in children with ASD.

## 3. Materials and Methods

This study was a randomized controlled trial conducted between September 2024 and November 2024 at the Oasi Research Institute–IRCCS in Troina, Italy, a research center specializing in the diagnosis, treatment, and rehabilitation of intellectual and developmental disabilities. Ethical approval was obtained from the Local Ethics Committee of the Oasi Research Institute–IRCCS (approval code: 2023/15/03/CE-IRCCS-OASI/253), and written informed consent was obtained from the parents or legal guardians of all participants before enrollment. The study adhered to the ethical principles outlined in the Declaration of Helsinki.

Participants were recruited from patients referred to the diagnostic and rehabilitation services of the Oasi Research Institute–IRCCS.

The inclusion criteria were age between 10 and 14 years, a confirmed Level 1 ASD diagnosis by expert neuropsychiatrists, following DSM-5 criteria [18], based on clinical evaluation and the use of standardized tests such as the Childhood Autism Rating Scale (CARS) [19], Autism Diagnostic Observation Schedule (ADOS-2) [20], and Autism Diagnostic Interview-Revised (ADI-R) [21]. Poor oral health was assessed using the oral health assessment tool (OHAT) [22], with a total score ranging from 3 to 16. The OHAT scale was administered by a chairside assistant specifically trained in its use, under the direct supervision of a dentist with longstanding experience in treating children with ASD. This approach was chosen to ensure a standardized and replicable assessment process. The exclusion criteria included patients with good oral health, defined as an OHAT score between 0 and 2, and the presence of psychiatric or neurological comorbidities.

Of the initial 83 patients screened, 19 were excluded due to good oral health, resulting in a final study sample of 64 participants. The demographic characteristics of the sample are summarized in Table 1.

### Procedure

**Figure 1:**
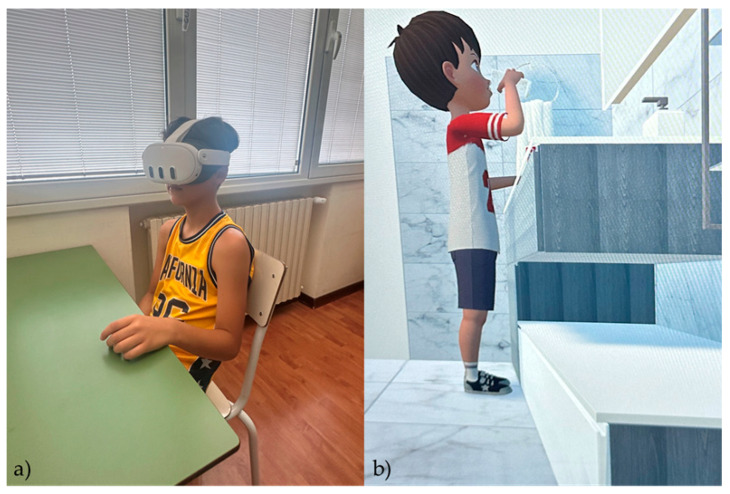
(**a**) A child involved in the study wearing oculus meta quest 3 VR headset; (**b**) virtual domestic bathroom setting and the avatar used to illustrate the correct oral hygiene routine.

In our study, we employed two different teaching methods for at-home oral hygiene:

1. Traditional oral hygiene instructions for learning correct at-home oral hygiene techniques: during the oral hygiene instruction session at the Dentistry Department of the Oasi Research Institute, the chairside dental assistant (S.L. or M.B.) demonstrated proper brushing techniques using a mouth model, as well as the correct use of mouthwash and dental floss.
2. Virtual reality (VR) “Domestic Bathroom” environment with an avatar, using VR headsets: during the oral hygiene instruction session at the Dentistry Department of the OASI MARIA SS IRCCS, the patient is asked to wear a VR headset (model: oculus meta quest 3), immersing them in a virtual domestic bathroom setting, where an avatar illustrates the correct oral hygiene maneuvers (Figure 1).

The VR experience was delivered through a Meta Quest 3 headset (Meta Platforms Inc. (Menlo Park, CA, USA); per-eye resolution: 2064 × 2208 pixels; field of view: ~110°; refresh rate: up to 120 Hz), equipped with advanced environmental and motion-tracking sensors, including 2 RGB cameras and 4 infrared cameras. The virtual scenario was developed in-house using Unity (version 2021.3.23f1) and featured a 3D bathroom environment in which a humanoid avatar demonstrated correct brushing and flossing techniques. The environment was designed to be visually repetitive and observational, to reduce sensory load, and facilitate learning in ASD children. Caregivers were instructed to supervise and support their child’s oral hygiene routine at home, particularly between training sessions and after meals. Although children were encouraged to brush and floss independently, when possible, autonomy was not mandatory. No formal logbook or structured record of daily hygiene practices was collected; therefore, adherence in the home setting was monitored indirectly through caregiver feedback and post-intervention assessments.

The oral health status of the patients, before and after the two interventions, was assessed using the OHAT scale administered by the chairside dental assistant.

The same dentist, having several years of experience treating children with ASD, assessed patients’ oral health status before and after two interventions using the OHAT scale. Both approaches (traditional oral hygiene instructions and the VR “domestic bathroom” environment) were administered at the Complex Operative Unit of Special Dentistry for Developmental Age at the Oasi Research Institute–IRCCS in Troina, Italy.

Initially, the entire sample of 83 patients underwent an oral health evaluation through a dental examination and the completion of the OHAT scale for a quantitative approach. Subsequently, all patients received treatment from the same dentist who performed tartar removal and addressed any existing dental caries, bringing each patient to a good oral health condition.

At this point, the 64 patients with an oral health status higher than two according to the OHAT scale were randomly divided into two groups of 32 patients each:

- Group 1: Received traditional oral hygiene instructions for learning correct at-home oral hygiene techniques;
- Group 2: Received the VR “Domestic Bathroom” environment intervention with an avatar through VR headsets.

**Figure 2:**
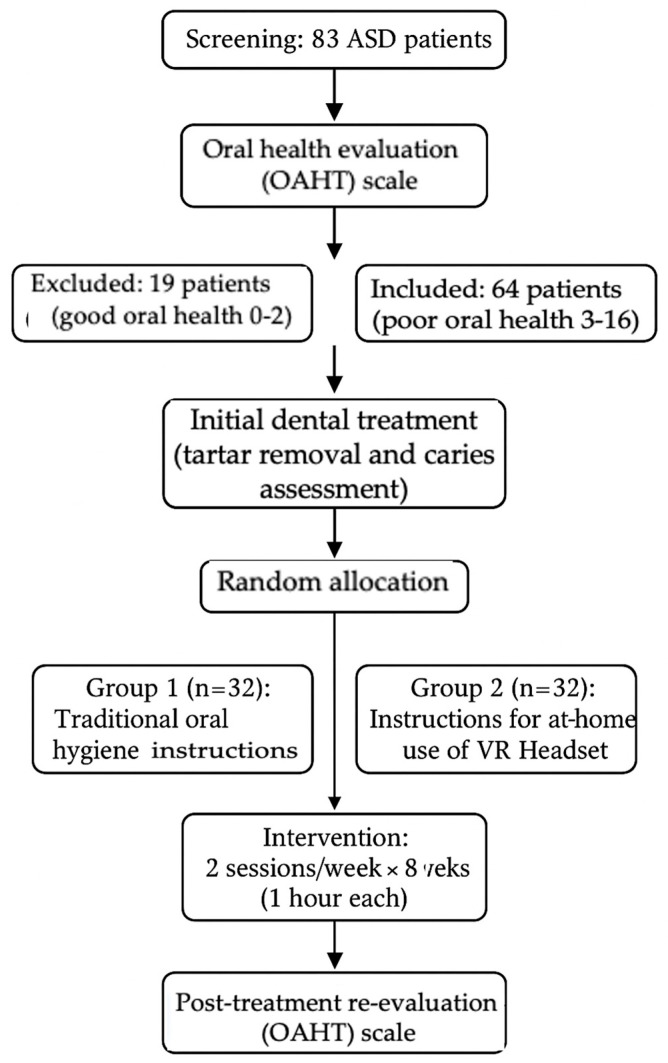
Flowchart of the study design. A total of 83 patients with Autism Spectrum Disorder (ASD) underwent oral health evaluation using the Oral Health Assessment Tool (OHAT) Scale. Nineteen patients with good oral health (score 0–2) were excluded, while 64 patients with poor oral health (score 3–16) received initial dental treatment, including tartar removal and caries assessment. Participants were then randomly allocated to two groups: Group 1 (*n* = 32), receiving traditional oral hygiene instructions, and Group 2 (*n* = 32), receiving instructions for at-home use of a Virtual Reality (VR) headset. Both groups participated in two one-hour sessions per week for eight weeks. Post-treatment oral health was reassessed using the OHAT Scale.

The hygiene therapy sessions for both groups were conducted twice a week for eight weeks, with each session lasting about one hour. At the end of the two-month period, both groups were re-evaluated using the OHAT scale (Figure 2).

Data analysis was performed using an unpaired t-test comparing Group 1 versus Group 2 in order to analyze pre- and post-intervention outcomes. The assumption of normality was assessed with the Kolmogorov–Smirnov test. All statistical analyses were carried out with IBM SPSS Statistics version 26.

## 4. Results

All 64 participants (32 in each group) completed the 8-week intervention period, and there were no dropouts. No significant compliance issues were reported in either group. The baseline characteristics of both groups, including age and sex distribution, were statistically comparable. The main results of the descriptive statistics are reported in Table 2.

Observational feedback indicated that participants in the VR group displayed higher levels of enthusiasm and engagement during training sessions. Caregivers of children in the VR group reported that their children were more willing to practice oral hygiene routines at home, while some caregivers in the control group had difficulties in maintaining their children’s interest in oral hygiene.

The *t*-test did not reveal any statistically significant differences between the two groups prior to the preventive intervention. However, following the preventive intervention, the t-test indicated a statistically significant difference between the group that received the VR-based preventive approach and the group that received the traditional preventive approach [t (62) = 10.4; *p* < 0.001] (see Table 3). This was driven by significantly better scores on the OHAT scale, underscoring an improvement in oral hygiene (mean ± SE; 7.6 ± 1.8 vs. 3.3 ± 1.4).

The response to the different types of preventive intervention does not appear to be influenced by sex.

## 5. Discussion

The findings of this study confirm that VR-based oral hygiene education can be a valuable tool for improving oral health outcomes in children with ASD. The greater reduction in OHAT scores in the VR group suggests that immersive, interactive learning environments are more effective than traditional instruction methods in reinforcing proper oral hygiene techniques. These results align with previous research demonstrating that children with ASD often respond better to technology-assisted interventions than to conventional teaching approaches [12,13,14].

Maintaining adequate oral hygiene is often a challenge for children with ASD due to sensory sensitivities, difficulties with fine motor coordination, and resistance to changes in routine [2]. Studies have shown that children with ASD are at a higher risk for dental caries, periodontal disease, and oral trauma due to a combination of behavioral, sensory, and dietary factors [2,3]. Additionally, barriers such as difficulties in communicating pain or discomfort, anxiety toward dental visits, and reduced access to specialized care can contribute to worsening oral health over time [8].

This study reinforces the need for tailored, multisensory educational tools for children with ASD, given their specific learning styles and sensory profiles. In this context, VR technology appears to be a promising and engaging alternative to traditional didactic approaches.

Traditional educational methods, such as printed materials or verbal instructions, may be less effective for children with ASD, who often require visual, structured, and interactive learning experiences to retain new information [17]. In this context, VR-based approaches can bridge the gap, offering a predictable, engaging, and controlled environment where children can learn at their own pace.

The results of our trial confirm and extend existing evidence suggesting that virtual reality (VR) is a powerful and versatile tool in autism interventions, not only in behavioral training, social skills, and therapeutic contexts, as shown in previous studies [23], but also in promoting engagement and adherence to oral hygiene routines in children with ASD. The immersive nature of VR helps reduce external distractions while providing real-time feedback, making it a highly effective method for skill acquisition in neurodivergent populations [15].

Several studies have demonstrated that VR-based interventions can improve self-care skills, executive functioning, and adaptive behaviors in children with ASD [10,11,12]. The success of VR in teaching daily living skills suggests that its application to oral hygiene training is both logical and evidence-supported. In our study, the VR-based training program significantly improved oral hygiene habits, as reflected in the greater reduction in OHAT scores compared to traditional instruction.

These findings reinforce the idea that children with ASD may benefit from multimodal, immersive learning experiences rather than conventional didactic methods [9]. The interactive nature of VR allows children to repeatedly practice brushing and flossing techniques in a non-threatening, gamified environment, helping to establish positive behavioral patterns over time [24,25].

Beyond the measurable improvements in oral hygiene, qualitative feedback from caregivers and observations during the study suggest that children in the VR group displayed higher motivation and engagement compared to those in the control group.

Parents in the VR group reported that their children were more willing to participate in oral hygiene routines at home. Resistance to brushing decreased over time as children gained confidence through VR training.

The gamification elements and interactive feedback kept children motivated and attentive during learning sessions. This increased engagement aligns with existing research indicating that gamification and immersive learning can enhance motivation and skill retention in children with ASD [9]. Furthermore, VR exposure may help desensitize children to sensory discomfort associated with brushing, addressing one of the most common barriers to effective oral hygiene in ASD populations [14,23].

The results of this study suggest that VR-based oral hygiene education could be incorporated into broader intervention strategies for children with ASD. Given the success of VR in educational and therapeutic applications, its integration into home-based training programs could offer a practical and scalable solution for improving long-term oral health outcomes in this population [9,23,26].

Future research should explore the long-term effectiveness of VR-based oral hygiene training beyond the 8-week study period or the feasibility of remote VR training models, allowing caregivers to implement VR-based oral hygiene education at home with minimal professional supervision.

Additionally, studies should investigate the cost-effectiveness of VR-based interventions compared to traditional education methods, considering their potential to reduce long-term dental complications and associated healthcare costs.

Even if this study provides strong evidence for the efficacy of VR-based oral hygiene education, several limitations should be considered, such as the variability in sensory sensitivities among children with ASD, which may affect their receptiveness to VR training.

Another limitation is the lack of a structured monitoring system to assess the consistency of home-based oral hygiene routines. While caregivers were instructed to supervise the activities, the absence of a formal log or diary limits the objectivity of adherence measurement in the home setting. Additionally, we acknowledge that using only the OHAT scale—while advantageous for its simplicity and tolerability—may have limited the clinical specificity of our oral health assessment compared to more technical indices. Future studies may consider combining OHAT with additional objective oral hygiene measures, where feasible.

The sample size, while adequate for statistical comparisons, should be expanded in future studies to increase generalizability.

## 6. Conclusions

The findings of this study demonstrate that VR-based oral hygiene education significantly improves oral health outcomes in children with ASD. Compared to traditional pedagogical methods, VR training resulted in greater reductions in OHAT scores, higher engagement levels, and increased motivation for oral hygiene routines. These results suggest that VR technology could play a crucial role in bridging the gap between clinical recommendations and at-home oral hygiene practices in neurodivergent populations.

Given the increasing accessibility of affordable VR technology, the potential for widespread implementation of VR-based oral hygiene programs is promising. This study supports further exploration of digital interventions as a means to enhance self-care skills and health outcomes in individuals with ASD.

## Figures and Tables

**Table 1:** Demographic characteristics of the sample.

Characteristic	Total Sample (*n* = 64)	Group 1 (*n* = 32)	Group 2 (*n* = 32)
Female (F)	30	14	16
Male (M)	34	18	16
Age (Minimum–Maximum)	10–14	10–14	10–14
Mean (SD)	12 (1.5)	12.1 (1.4)	12.1 (1.5)
Age distribution	Frequency (%)	–	–
10	13 (20.3%)	–	–
11	12 (18.8%)	–	–
12	10 (15.6%)	–	–
13	14 (21.9%)	–	–
14	15 (23.4%)	–	–

**Table 2:** Main results of the descriptive statistics.

Measure	Group 1 OHAT-Pre	Group 1 OHAT-Post	Group 2 OHAT-Pre	Group 2 OHAT-Post
Mean	10.5	7.6	11.0	3.3
Median	10.0	8.0	11.0	3.0
Variance	3.4	3.3	4.2	2.0
Standard Deviation	1.8	1.8	2.1	1.4

Group 1: Traditional pedagogical approach; Group 2: VR intervention; OHAT-pre: Assessment before the intervention; OHAT-post: Assessment after the intervention.

**Table 3:** Sample t-test differences between pre-test and post-test for Group 1 vs. Group 2.

Comparison	t	df	*p*-Value	Mean Difference	Std. Error of the Difference	95% CI (Lower)	95% CI (Upper)
G1 vs. G2 (OHAT_pre)	−0.957	62	0.342	−0.469	0.490	−1.448	0.510
G1 vs. G2 (OHAT_post)	10.423	62	<0.001	4.250	0.408	3.435	5.065

df: degrees of freedom.

## Data Availability

The de-identified participant data that support the findings of this study are available from the corresponding author upon reasonable request, pending approval of the request by the Ethics Committee of IRCCS Oasi Maria SS. to ensure compliance with ethical and legal obligations.

## References

[1] Sami W., Ahmad M.S., Shaik R.A., Miraj M., Ahmad S., Molla M.H. (2023). Oral health statuses of children and young adults with autism spectrum disorder: An umbrella review. J. Clin. Med..

[2] Ferrazzano G.F., Salerno C., Bravaccio C., Ingenito A., Sangianantoni G., Cantile T. (2020). Autism spectrum disorders and oral health status: Review of the literature. Eur. J. Paediatr. Dent..

[3] Bernath B., Kanji Z. (2021). Exploring barriers to oral health care experienced by individuals living with autism spectrum disorder. Can. J. Dent. Hyg..

[4] Siemann J.K., Veenstra-VanderWeele J., Wallace M.T. (2020). Approaches to understanding multisensory dysfunction in autism spectrum disorder. Autism Res..

[5] Fallea A., Vetri L., L’Episcopo S., Bartolone M., Zingale M., Di Fatta E., d’Albenzio G., Buono S., Roccella M., Elia M. (2024). Oral Health and Quality of Life in People with Autism Spectrum Disorder. J. Clin. Med..

[6] Fallea A., Zuccarello R., Roccella M., Quatrosi G., Donadio S., Vetri L., Calì F. (2022). Sensory-adapted dental environment for the treatment of patients with autism spectrum disorder. Children.

[7] Prynda M., Pawlik A.A., Niemczyk W., Wiench R. (2024). Dental Adaptation Strategies for Children with Autism Spectrum Disorder—A Systematic Review of Randomized Trials. J. Clin. Med..

[8] Petersen P.E., Bourgeois D., Ogawa H., Estupinan-Day S., Ndiaye C. (2005). The global burden of oral diseases and risks to oral health. Bull. World Health Organ..

[9] Zerman N., Zotti F., Chirumbolo S., Zangani A., Mauro G., Zoccante L. (2022). Insights on dental care management and prevention in children with autism spectrum disorder (ASD). What is new?. Front. Oral Health.

[10] Stein C., Santos N.M.L., Hilgert J.B., Hugo F.N. (2018). Effectiveness of oral health education on oral hygiene and dental caries in schoolchildren: Systematic review and meta-analysis. Community Dent. Oral Epidemiol..

[11] Flannery K.A., Wisner-Carlson R. (2020). Autism and education. Child Adolesc. Psychiatr. Clin..

[12] Parsons S., Cobb S. (2016). State-of-the-art of virtual reality technologies for children on the autism spectrum. Technology and Students with Special Educational Needs.

[13] Lorenzo G., Lledó A., Pomares J., Roig R. (2016). Design and application of an immersive virtual reality system to enhance emotional skills for children with autism spectrum disorders. Comput. Educ..

[14] Ke F., Im T. (2013). Virtual-reality-based social interaction training for children with high-functioning autism. J. Educ. Res..

[15] Zhao J., Zhang X., Lu Y., Wu X., Zhou F., Yang S., Wang L., Wu X., Fei F. (2022). Virtual reality technology enhances the cognitive and social communication of children with autism spectrum disorder. Front. Public Health.

[16] Al Kheraif A.A., Adam T.R., Wasi A., Alhassoun R.K., Haddadi R.M., Alnamlah M. (2024). Impact of Virtual Reality Intervention on Anxiety and Level of Cooperation in Children and Adolescents with Autism Spectrum Disorder during the Dental Examination. J. Clin. Med..

[17] Amin F., Waris A., Syed S., Amjad I., Umar M., Iqbal J., Gilani S.O. (2024). Effectiveness of Immersive Virtual Reality Based Hand Rehabilitation Games for Improving Hand Motor Functions in Subacute Stroke Patients. IEEE Trans. Neural Syst. Rehabil. Eng..

[18] Edition F. (2013). Diagnostic and statistical manual of mental disorders. Am. Psychiatr. Assoc..

[19] Schopler E., Reichler R.J., Renner B.R. (1988). The Childhood Autism Rating Scale (CARS) Western Psychological Services.

[20] Lord C., Rutter M., DiLavore P., Risi S., Gotham K., Bishop S. (2012). Autism Diagnostic Observation Schedule–2nd Edition (ADOS-2).

[21] Rutter M., Le Couteur A., Lord C. (2003). Autism Diagnostic Interview-Revised.

[22] Chalmers J.M., King P.L., Spencer A.J., Wright F.A.C., Carter K.D. (2005). The oral health assessment tool—Validity and reliability. Aust. Dent. J..

[23] Yang X., Wu J., Ma Y., Yu J., Cao H., Zeng A., Fu R., Tang Y., Ren Z. (2025). Effectiveness of Virtual Reality Technology Interventions in Improving the Social Skills of Children and Adolescents With Autism: Systematic Review. J. Med. Internet Res..

[24] Krzysztofiak A., Chiappini E., Venturini E., Gargiullo L., Roversi M., Montagnani C., Bozzola E., Chiurchiu S., Vecchio D., Castagnola E. (2021). Italian consensus on the therapeutic management of uncomplicated acute hematogenous osteomyelitis in children. Ital. J. Pediatr..

[25] Caliendo M., Di Sessa A., D’Alterio E., Frolli A., Verde D., Iacono D., Romano P., Vetri L., Carotenuto M. (2021). Efficacy of neuro-psychomotor approach in children affected by autism spectrum disorders: A multicenter study in Italian pediatric population. Brain Sci..

[26] Didehbani N., Allen T., Kandalaft M., Krawczyk D., Chapman S. (2016). Virtual reality social cognition training for children with high functioning autism. Comput. Hum. Behav..

